# Contribution of *Balkan Medical Journal* to the development and dissemination of medical science in the Balkans

**DOI:** 10.4274/balkanmedj.2018.3.0001

**Published:** 2018-05-28

**Authors:** Zafer Koçak, Necdet Süt, Mustafa İnan

**Affiliations:** 1 Department of Radiation Oncology, Trakya University School of Medicine, Edirne, Turkey; 2 Department of Biostatistics and Informatics, Trakya University School of Medicine, Edirne, Turkey; 3 Department of Pediatric Surgery, Trakya University School of Medicine, Edirne, Turkey

*Balkan Medical Journal* is an institutional initiative designed to disseminate scientific knowledge produced by long-established human communities residing in the Balkan Peninsula (1). Since its conception, the journal management has aimed to publish eminent research from the Balkans (2). Today, we consider that the attention of scientists from the Balkans to our journal has increased. In this editorial, we aimed to analyze the relationship of *Balkan Medical Journal* with its scientific partners from the Balkans.

In a previous analysis, we presented information regarding the sources of citations to *Balkan Medical Journal* (3). Our results indicated that unfortunately, *Balkan Medical Journal* was cited by other journals and scientists from Balkan countries at a rate far below our expectations. Nonetheless, this finding encouraged us to analyze the data from a different perspective. Therefore, we aimed to assess the number of articles from the Balkans that have been published in *Balkan Medical Journal* as well as the possible implications of the research findings. We used ScholarOne Manuscript^TM^ (https://mc04.manuscriptcentral.com/balkanmedj) to retrieve data from 2014 to 2017. Moreover, we used data from Clarivate Analytics InCites to assess the impact of retrieved publications (https://clarivate.com/products/incites/).

When the journal’s acceptance rates were analyzed according to years and regions, the acceptance rates of articles from the Balkans were consistently higher than the overall acceptance rates. Furthermore, the acceptance rates for countries outside the Balkans remained below the overall acceptance rate until 2016 but significantly increased since 2017, exceeding the overall acceptance rate. Conversely, the acceptance rates for articles from Turkey submitted after 2015 were below the average acceptance rates (Figure 1).

The number of articles submitted from the Balkans in 2017 (n=137) was 5.5 times the number of articles submitted in 2014 (n=25). Interestingly, the increase in the submission and acceptance rates (published) from the Balkans over the years was remarkable. The submission (number of submission from Balkans/total number of submission ×100) and acceptance (number of accepted manuscripts from Balkans/total number of accepted manuscripts ×100) rates from Balkan countries increased from 2.43% in 2014 to 8.09% in 2017 and from 3.96% in 2014 to 10.68% in 2017, respectively (Figure 2). These results indicate that the increase in the acceptance rate was higher than that in the submission rate. Indeed, we consider that the articles from the Balkans should be represented at a higher rate in *Balkan Medical Journal* cares, as reflected by our findings. Between 2014 and 2017, the average acceptance rate from the Balkans was 8.7%, while the average rate of citations from the Balkans to our journal was only 4.8% (Figure 2), indicating that *Balkan Medical Journal* has a greater potential to be cited by the Balkans.

The distribution of accepted articles by region is shown in Figure 3. The proportion of publications from countries other than Turkey increased from 10.89% in 2014 to 31.07% in 2017. Approximately, one-third of this increase was contributed by the Balkans, which may be attributable to the increased international visibility and impact of the journal.

We used Clarivate Analytics InCites data to assess the impact of publications from the Balkans. For a more accurate evaluation, we considered data until 2016 (2011–2016). The results of this analysis are summarized in Table 1. Approximately 81% (39/48) of these publications from the Balkans in this period were from Bulgaria, Serbia, and Romania. This finding indicates that we should make the journal more recognizable to other Balkan countries as well. Approximately 48% (23/48) of these publications were cited once or more. According to category-normalized citation impact analysis data, articles from Croatia, Serbia, and Montenegro showed higher scores than articles from other Balkan countries.

In conclusion, a 5.5-fold increase in the number of articles submitted from the Balkans is a favorable indication for the future. Notably, the acceptance rate of articles from the Balkans has now reached 10.68%, however the citation rate of these articles highlights the importance of increasing the quality of research. Overall, our findings indicate the need to gain broader recognition especially in the Balkans and also other countries.

## Figures and Tables

**Table 1:**
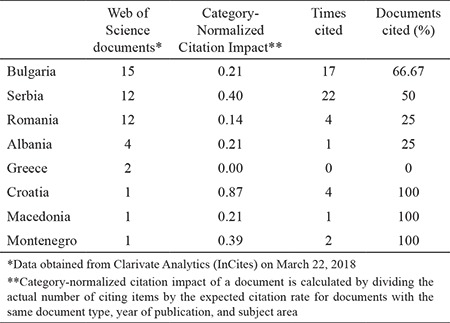
The partnership of Balkan Medical Journal’s with Balkan countries between 2011 and 2016

**Figure 1:**
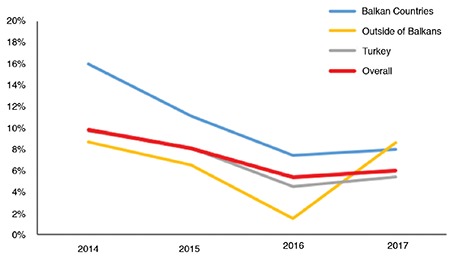
Acceptance rates (e.g., the number of accepted manuscripts from the Balkans/total number of submitted manuscripts from the Balkans) between 2014 and 2017 stratified according to regions. The acceptance rates of manuscripts from the Balkans were higher than the overall acceptance rate for all years (data obtained from ScholarOne Manuscripts^TM^ on April 1, 2018).

**Figure 2:**
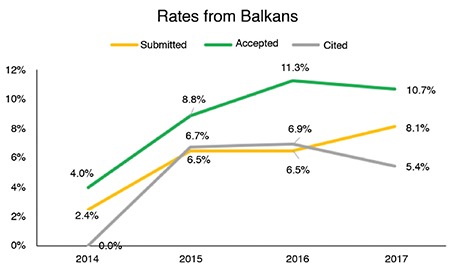
The rates of submission, acceptance, and citations from the Balkans are shown. The submission (number of submission from Balkans/total number of submission ×100) and acceptance (number of accepted manuscripts from Balkans/total number of accepted manuscripts ×100) rates from the Balkans increased from 2.43% in 2014 to 8.09% in 2017 and 3.96% in 2014 to 10.68% in 2017, respectively. While the average acceptance rate from the Balkans was 8.7%, and the average citation rate (number of citations from Balkans/total number of citations ×100) from the Balkans to our journal was only 4.8%.

**Figure 3:**
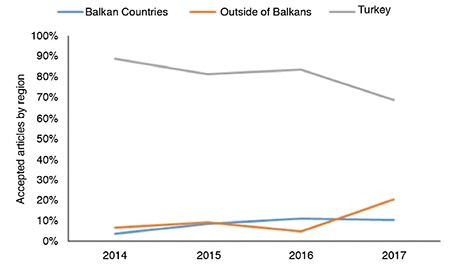
Distribution of accepted articles by region. The rate of publications from countries other than Turkey increased from 10.89% in 2014 to 31.07% in 2017. The Balkans contributed approximately one-third of this increase (data obtained from ScholarOne Manuscripts^TM^ on April 1, 2018).
